# Trophic structure modulates community rescue following acidification

**DOI:** 10.1098/rspb.2019.0856

**Published:** 2019-06-12

**Authors:** Graham Bell, Vincent Fugère, Rowan Barrett, Beatrix Beisner, Melania Cristescu, Gregor Fussmann, Jesse Shapiro, Andrew Gonzalez

**Affiliations:** 1 Biology Department, McGill University, 1205 ave docteur-Penfield, Montreal, Quebec, Canada H3A 1B1; 2 Département des sciences biologiques, Université du Québec à Montréal, 141 ave du Président-Kennedy, Montreal, Quebec, Canada H2X 1Y4; 3 Redpath Museum, McGill University, 859 rue Sherbrooke O, Montreal, Quebec, Canada H3A 0C4; 4 Département de sciences biologiques, Pavillon Marie-Victorin, 90 ave Vincent-d'Indy, Montreal, Quebec, Canada H2 V 2S9

**Keywords:** phytoplankton, zooplankton, acidification, foodweb, community rescue, mesocosm experiment

## Abstract

Community rescue occurs when a community that experiences lethal stress persists only through the spread of rare types, either genotypes or species, resistant to the stress. Rescue interacts with trophic structure because physical stress experienced by a focal assemblage within the community may also be experienced by its predators and prey. In general, trophic structure will facilitate rescue only when a stress has a less severe effect on a focal assemblage than on its predators. In other circumstances, when stress affects prey or has only a weak effect on predators, trophic structure is likely to hamper rescue. We exposed a community of phytoplankton and zooplankton derived from a natural lake to acidification in outdoor mesocosms large enough to support trophically complex communities. Rescue of the phytoplankton from severe acidification was facilitated by prior exposure to sublethal stress, confirming previous results from microcosm experiments. Even communities that have previously been less highly stressed were eventually rescued, however, because their zooplankton predators were more sensitive to acidification and became extinct. Our experiment shows how community rescue following severe stress is modulated by the differential effect of the stress relative to trophic level.

## 1. Introduction

Organisms can often survive any particular stress by modifying their morphology, physiology or behaviour. If the stress is so severe that these plastic responses are inadequate, the whole population will become extinct unless some rare type which is constitutively resistant or has greater plasticity spreads in time. Laboratory experiments with single-species populations have shown how this process of evolutionary rescue is governed by genetic diversity, prior exposure to sublethal stress and dispersal in metapopulations [1–4]. A similar process of rescue may act at the level of entire ecological communities. A community (for our purposes) consists of all the organisms living in a particular place at a particular time. At a subsequent time, this community experiences the stress brought about because the conditions of life at this place change. The community may survive the stress because all its constituent species populations respond equally to undergo rescue through plastic or genetic modification, such that the species composition of the antecedent community is preserved unchanged. More generally, species will respond differently and consequently species composition will change, through the increase in abundance of resident or immigrant species better able to withstand the stress and the decrease of other species. This is similar to the process of evolutionary rescue in an asexual population caused by natural selection acting on standing genetic variation or novel mutation. It may likewise result in the preservation of some overall features of the antecedent community (such as productivity or trophic structure) even though many lineages leave few or no descendants. This is the process that we term ‘community rescue'. It differs from succession and resilience in that it implies recovery without environmental amelioration.

Community rescue may be brought about by physiological and developmental processes (plasticity), evolutionary processes (adaptation of species through natural selection) and ecological processes (changes in composition caused by species sorting). A complete accounting of plastic, evolutionary and ecological processes and their interactions (for example, species may differ in the plasticity they express or evolve) cannot be provided by an observational survey and instead requires a reciprocal transplant experiment or a detailed population pedigree [5,6] (M.D. Jewell & G.B. 2018, unpublished data). Moreover, the term ‘rescue', as we have defined it, implies that some general criterion (such as productivity or trophic structure) must be specified in advance; it may be only partially satisfied, and, whether fully or partially satisfied, does not imply that any other criterion would lead to the same conclusion. These considerations lead to awkward questions. As an extreme example, suppose that all the species in the antecedent community become extinct following the stress and are replaced by resistant immigrants. Granted that some measure of productivity (say) has been restored, in what meaningful sense can the antecedent community be said to have been ‘rescued' when none of its lineages has survived?

These difficulties can be minimized by investigating rescue in closed communities (such as laboratory populations of microbes that feed by absorbing organic substrates) and using optical density as a measure of overall biomass. An experiment of this kind, using highly diverse microbial communities, showed that community rescue was modulated by the same factors as evolutionary rescue in single-species populations, suggesting that it can be understood by a simple extension of the same principles [7]. This approach simplifies the problem to the point where it can be solved, but in turn leads to two cogent objections. The first objection is that natural communities often exhibit complex trophic interactions among predators, prey, parasites, detritivores and other kinds of organism that are lacking in laboratory cultures. The second objection is that ecological and evolutionary processes often vary with scale, so that results from laboratory microcosms cannot be legitimately extended to lakes or seas whose volume is many orders of magnitude greater. These two objections are, to some extent, complementary: trophic structure with an extensive ecological division of labour between different ways of life requires a correspondingly large and extensive community. Both trophic structure and spatial scale must be incorporated into a realistic and generally applicable theory of evolutionary rescue.

In this report, we first present a simple model illustrating how trophic structure might modulate community rescue. We then describe an experiment in which the response to lethal physical stress was studied in trophically structured metacommunities maintained in mesocosms whose volume of 1000 l was intermediate on a log scale between a laboratory microcosm of 100 µl and a small lake with a radius of 1 km and a mean depth of 3 m.

## 2. Methods: effects of trophic structure on community rescue

### (a) Trophic structure and stress

**Figure 1.**
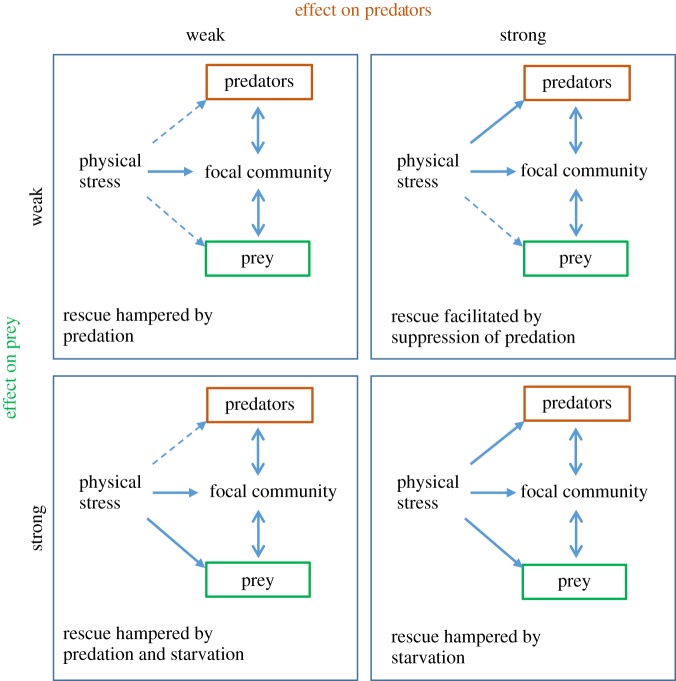
The effect of a physical stress on a focal population within a trophically structured community. The double-sided arrows indicate that trophic interactions can either exacerbate or ameliorate the effect of the stressor. (Online version in colour.)

A severe physical stress experienced by some focal population or community may also be experienced by its predators or its prey or both. Whether or not it is rescued depends both on its innate ability to acquire resistance and on the response of the other kinds of organism (figure 1). If the stress has only a weak effect on the predators, then its effect on the focal population will be exacerbated by predation; conversely, if it has a strong effect on prey, its effect will be exacerbated by starvation; worst of all is a stress that has a weak effect on predators and a strong effect on prey. It is only the combination of a strong effect on predators and a weak effect on prey that will alleviate the stress experienced by the focal population and render rescue more likely to occur. Community rescue in trophically structured communities where predators might drive prey to extinction has been studied theoretically by Kovach-Orr & Fussmann [8] and Yamamichi & Miner [9]. Fussmann & Gonzalez [10], Northfield & Ives [11] and Osmond *et al*. [12] have analysed models of rescue in trophically structured communities experiencing deteriorating conditions of growth. Tseng & O'Connor [13] found that predation by *Chaoborus* altered the response of laboratory populations of *Daphnia* to mildly elevated temperature. Thibodeau *et al*. [14] used in-lake mesocosms to study the effect of acidification on phytoplankton and zooplankton communities. With these exceptions, however, there seems to be no previous experimental work specifically directed towards the rescue of trophically structured communities exposed to severe physical stress.

### (b) Community model

We set up a simple mathematical model as a framework for testing hypotheses and interpreting our results. The simplest trophically structured community would comprise only two kinds of organism: primary producers (such as phytoplankton) and the herbivores that consume them (such as zooplankton). This situation can be represented by a modified Lotka–Volterra model in which both phytoplankton and zooplankton have susceptible and resistant types. The model iterates the abundance of individuals susceptible or resistant to a stressor within two trophic compartments: the phytoplankton (*P*_sus_ and *P*_res_; total *P*) and zooplankton (*Z*_sus_ and *Z*_res_; total *Z*). In the absence of either zooplankton or stress, the finite rate of increase of phytoplankton is *a*, which is reduced by stress by an amount *s_P_* in susceptible types and *r_P_* in resistant types. Similarly, the death rate of zooplankton in the absence of stress is *d*, which is increased by *s_Z_* in susceptible and *r_Z_* in resistant types. When both phytoplankton and zooplankton are present, the death rate of phytoplankton when encountering zooplankton is an attack coefficient *x*, and the efficiency of conversion of phytoplankton to zooplankton is *c*. Finally, the phytoplankton population is regulated logistically, with carrying capacity *Q* and a coefficient α expressing the strength of density-dependence. The recursion equations are then:

$$\begin{array}{rl} & \mathrm{phytoplankton}:\;\mathrm{susceptible}\;\;P_{\mathrm{sus}}(t+1)=(1+a-s_{P})\times P_{\mathrm{sus}}(t)\times \exp(\alpha \;\times (Q-P(t))/Q))-x\times P_{\mathrm{sus}}(t)\times Z(t)\\ & \;\;\;\;\mathrm{resistant}\;\;P_{\mathrm{res}}(t+1)=(1+a-r_{P})\times P_{\mathrm{res}}(t)\times \exp(\alpha \;\times (Q-P(t))/Q))-x\times P_{\mathrm{res}}(t)\times Z(t)\\ & \mathrm{zooplankton}:\;\;\mathrm{susceptible}\;Z_{\mathrm{sus}}(t+1)=(1-d-s_{Z})\times Z_{\mathrm{sus}}(t)+c\times x\times P(t)\times Z_{\mathrm{sus}}(t)\\ & \;\;\;\;\mathrm{resistant}\;\;Z_{\mathrm{res}}(t+1)=(1\text{–}d-r_{Z})\times Z_{\mathrm{res}}(t)+c\times x\times P(t)\times Z_{\mathrm{res}}(t). \end{array}$$

This can be used to show how the likelihood that phytoplankton will be rescued after exposure to lethal stress will be reduced by two features of a trophically structured community: depressed abundance and a lower critical level of stress.

### (c) Depression of abundance by predation

If there are no zooplankton, the phytoplankton population approaches *P̂* = *Q*[1 + ln(1 + *a* − *s_P_*)/*α*], assuming that the frequency of resistant types is negligible. In the absence of stress, if *a* is not too large, this expression is roughly the ratio of density-independent to density-dependent forces, multiplied by a scaling factor: *P̂* ≈ (1 + *a*/*α*) *Q*. When zooplankton are introduced, there is a stable joint equilibrium at:

$$\begin{array}{rl} & \hat{P}=Q\{[1-\ln[(1+x\hat{Z})/(1+a-s_{P})]\}\\ & \hat{Z}=(1/x)\;\{\;(1+a-s_{P})\exp[\alpha \;\{Q-\hat{P})/Q]-1\} \end{array}$$

One way of expressing this result is that when zooplankton are present, phytoplankton abundance is depressed by a quantity *Q* ln[1 + *xẐ*], where *x* is an attack coefficient.

### (d) Critical level of stress

There is a critical level of stress *u_P_* for phytoplankton in the absence of zooplankton at which the population barely persists. This can be calculated by setting *P̂* = 0 and solving to yield u_P_* = 1 + *a* − e^−^*^α^*. If *s_P_* > u_P_*, the population declines to extinction unless rescued by the spread of resistant types. If zooplankton are present, this condition becomes more onerous because of the additional death rate imposed by predation: the critical level of stress *v_P_* when zooplankton are present is v_P_* = 1 + *a* − e^−^*^α^* [1 + *xẐ*]. The difference *δ*s_P_ between the critical level when zooplankton are absent and that when they are present is *δs_P_* = e^−^*^α^xẐ*, reflecting the additional destruction of phytoplankton by predation.

**Figure 2.**
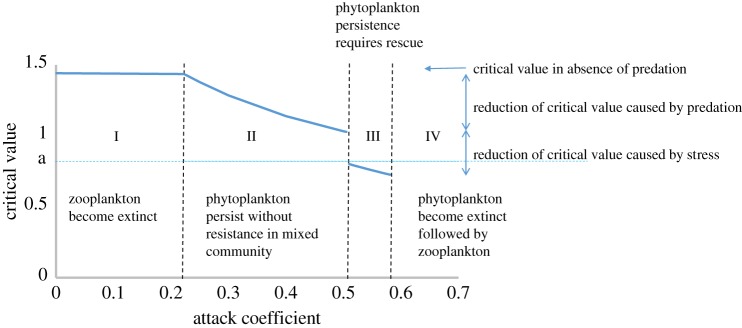
How the critical value of stress is reduced by predation. The critical value is the greatest value of stress that can be tolerated by the phytoplankton community, with or without the spread of resistant types. The *per capita* rate of increase of phytoplankton in the absence of stress and predation is 1 + *a*. (Online version in colour.)

The impact of predation on the phytoplankton is mediated by the attack coefficient *x*. When other parameters are fixed, the value of *x* governs the abundance of phytoplankton and zooplankton, and the critical value of stress. A community with a particular value of *x* occupies one of four distinct dynamic regimes, which are illustrated in figure 2.

I. Low attack success with *s_P_* < u_P_*. Zooplankton are unable to invade; susceptible phytoplankton persist at high abundance. The critical level of stress refers to its physical effect alone.
II. Moderate attack success with *s_P_* < v_P_*. Zooplankton become established, but susceptible phytoplankton can persist without the spread of resistance. Rescue is unnecessary. (Resistant types may nonetheless spread if they have greater relative fitness.) The critical value v_P_* falls as *x* increases because of the additional mortality caused by predation.
III. Moderate attack success with *s_P_* > v_P_* > *r_P_*. Rescue is necessary: the effect on susceptible types exceeds the critical level of stress, and phytoplankton persist only if resistant types replace susceptible types. The critical value falls abruptly between regimes II and III because of the abrupt increase in zooplankton abundance, caused by the increased abundance of phytoplankton once resistance has become fixed, and continues to fall as attack success increases.
IV. High attack success with v_P_* < *r_P_*. Rescue is ineffective: the effect of stress even on the resistant types exceeds the critical level, and consequently phytoplankton become extinct, followed by zooplankton.

Figure 2 is a numerical example illustrating these regimes. The decline in the critical value following regime I expresses the effect of predation on the likelihood of rescue.

## 3. Methods: experimental test of the effects of trophic structure

### (a) Experimental facility

The experiment reported here was conducted at the Large Experimental Array of Ponds (LEAP) at the Gault Nature Reserve, Mont St-Hilaire, Quebec. LEAP contains 96 circular tanks each with a capacity of 1100 l, which are used as mesocosms. They are arranged in groups of 8 around a common source of water and electrical power. The mesocosms are fed from an upstream reservoir and empty into a downstream holding pond.

### (b) Acid lakes

We used acidification as a physical stressor because the ecology of lakes acidified by mining or industrial emissions has been a major source of concern in many countries. In general, total phytoplankton biomass is little affected by moderate acidification to pH 5 or so, and may increase, for example by nitrogen fertilization derived from nitric acid [15]. Species composition may be altered, however, and diversity reduced: for example, the phytoplankton communities of many acidified lakes in North America are dominated by the dinoflagellate *Peridinium inconspicuum* [16], either because it is naturally resistant or because it is rejected by cladocerans. Many species of crustacean zooplankton and benthic invertebrates are depleted by moderate acidification [17]. The experimental evidence for an interaction between phytoplankton and zooplankton is equivocal: in acidified mesocosms from which zooplankton were excluded chlorophytes became dominant, even though *P. inconspicuum* was initially present, suggesting that although these chlorophytes are acid-tolerant, they are normally suppressed by predation, but the exclusion of zooplankton from mesocosms in a chronically acidified lake did not lead to an increase in the abundance of chlorophytes [18]. Thibodeau *et al*. [14] found evidence for genetic adaptation of chlorophytes to acidification during in-lake mesocosm experiments. Both phytoplankton and zooplankton communities may be reconstituted when pollution ceases and the lakes recover to their previous pH [19,20], although the recovery of severely acidified lakes is often slow and incomplete [21].

### (c) Metacommunity structure

Spatial structure may contribute to rescue through the movement of partially resistant individuals up a gradient of stress, creating the opportunity for selection to enhance the level of resistance. The mode of dispersal in metacommunities was an important factor governing the frequency of rescue among microbial communities in a laboratory microcosm experiment [7]. We expected dispersal to increase abundance in stressful conditions through the immigration of partially adapted individuals from somewhat less stressful mesocosms, thereby elevating the frequency of rescue when the community encountered lethal stress.

### (d) Base communities

The reservoir at LEAP is connected by a pipeline (diameter: 10 cm) to lac Hertel, a small mesotrophic lake on the Gault reserve about 100 m uphill and about 1 km distant. The inlet of the pipeline in the lake is about 50 m offshore and captures a representative sample of lake plankton which forms the base community for our experiments. The two main trophic compartments of this community are the primary producers (phytoplankton) and the animals that consume them (zooplankton). All LEAP ponds were filled and inoculated on 23/24 May 2017. A nutrient spike of 40 mg P (as KH_2_PO_4_ and K_2_HPO_4_) and 600 mg N (as KNO_3_) was added to each pond before inoculation to stimulate primary production. This nutrient supplement had the same molar N : P ratio as lac Hertel water. The pond communities were allowed to stabilize for two weeks before the experiment began on 5 June 2017.

### (e) Community monitoring

The mesocosms were monitored weekly. Water samples were collected from the upper 35 cm of the water column using integrated samplers made from tubing of 2.5 cm diameter. Chlorophyll a concentration was estimated using a Fluoroprobe (BBE Moldaenke) as a proxy for phytoplankton biomass. These measurements provided estimates of the concentrations of four different pigments corresponding to the relative abundance of cyanobacteria, chlorophytes, brown algae (mainly diatoms in Lac Hertel, but also including chrysophytes and dinoflagellates) and cryptophytes. Crustacean zooplankton abundance was measured in 2 l samples passed through a 64-µm mesh and resuspended in 95% ethanol. Preserved samples were scored under a low-power microscope and individuals assigned to ordinal categories (copepods and cladocerans). Water pH was measured *in situ* with a hand-held probe (YSI Inc., Yellow Springs, OH, USA). Adult hemipterans and the predatory larvae of beetles, dragonflies and *Chaoborus* were seen in the mesocosms but not counted.

### (f) Experimental design

**Figure 3.**
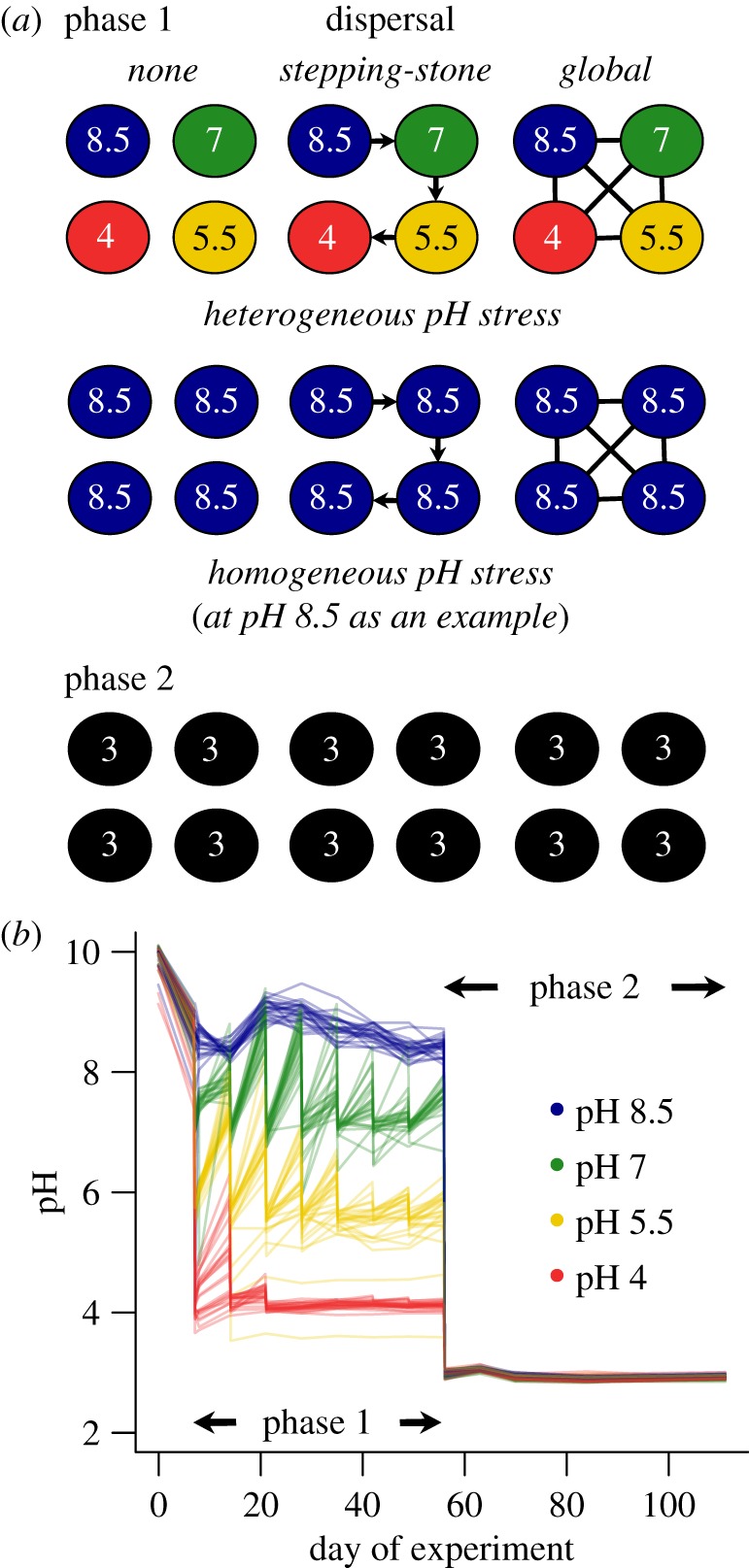
pH and dispersal treatments. (*a*) Design of our two-phase experiment. Circles represent mesocosms. Colours and numbers indicate pH treatments, while arrows illustrate the flow of water between ponds in metacommunities with dispersal. A subset of 6 out of 24 metacommunities are shown to illustrate the range of treatment combinations. Homogeneous metacommunities included all possible pH treatments; we show pH 8.5 as an example. Phase 2 also included four control ponds which were not acidified. (*b*) Pond pH over the course of the experiment. Each line represents an individual mesocosm, while colours distinguish pH treatments. (Online version in colour.)

We investigated the effect of severe physical stress on the community by conducting a press-pulse experiment in two phases. In phase 1, we imposed and maintained different levels of a stress, acidification, for a period of seven weeks from May to July 2017. During this phase, the experimental communities had the opportunity to respond to stress by physiological, ecological or evolutionary modifications. In phase 2, we applied a uniform stress to all mesocosms so severe that it was lethal to the base, unmodified community. This design enables us to estimate the effect of prior exposure to the stress on the incidence of rescue. The overall experimental design is illustrated in figure 3*a*.

### (g) Treatments

We manipulated pH and dispersal in the mesocosms in a factorial manner. The water of lac Hertel is at about pH 8.5. We created three levels of greater acidity by weekly titration with 10N HCl during phase 1, to create four treatments of pH 8.5, pH 7, pH 5.5 and pH 4. At the beginning of the experiment pH tends to return towards its original value of 8.5 during the week in all treatments although this return is slow and partial at pH 4 (figure 3*b*). Later in the experiment, ponds lost their buffering capacity and pH remained at the target level through the week. During phase 2, all ponds were acidified to pH 3 and maintained at this pH permanently without the need for further treatment (figure 3*b*). The experiment ended on 26 September 2017.

The mesocosms were arranged in groups of 4 to form metacommunities. A dispersal treatment was applied to each metacommunity: none (no transfer of material between mesocosms), global (1% from each mesocosm mixed in a pool and redistributed) or stepping-stone (1% transferred from a mesocosm at given pH to the mesocosm with pH one level lower, with 1% added to pH 8.5 mesocosms from the reservoir and 1% discarded from pH 4 mesocosms to the holding pond). There were two kinds of metacommunity structure: homogeneous metacommunities (in which all mesocosms were at the same pH) and heterogeneous metacommunities (which included all four pH treatments). The homogeneous metacommunities served as controls to estimate any additional effect on rescue of moving pre-adapted individuals up the pH gradient towards more acidified mesocosms.

The experiment thus had a factorial structure with 4 pH levels × 3 dispersal procedures × 2 metacommunity structures × 4 replicates = 96 mesocosms. We also set up four control ponds that were not acidified in phase 2 to identify seasonal effects on communities in the absence of physical stress. The experiment was set up on the ground as completely randomized blocks, each block being a metacommunity. We expected a main effect of pH for both kinds of metacommunity, generated by lower abundance at lower current pH in phase 1 because of the direct effect of acidification, and by higher abundance (or higher frequency of rescue) at lower prior pH in phase 2 because of prior adaptation to stress. We expected dispersal between mesocosms to increase abundance at low pH in phase 1, through the immigration of partially adapted individuals from somewhat higher pH, and we expected that this effect would persist in phase 2, because of the enhanced adaptation to low pH of communities that had previously received immigrants. This will generate a pH × dispersal interaction, with abundance being greatest for the combination of low pH and high dispersal. Given an effect of dispersal, we expected stepping-stone dispersal to be more effective than global dispersal, because it will specifically direct more individuals to any target community from the next most stressful treatment. These effects of dispersal should be observed in the heterogeneous but not in the homogeneous metacommunities; an effect observed in both would be attributable to some more general consequence of dispersal, such as larger effective population size.

### (h) Statistical analysis

Time series of phytoplankton and zooplankton abundance were analysed with separate generalized additive mixed models (GAMMs) fitted using R v. 3.5.0 [22] along with the package ‘mgcv' [23]. Before fitting the model, chlorophyll a concentration was log-transformed, crustacean zooplankton density was log(*x* + 1)-transformed and time (number of weeks after the first acid and dispersal treatments) was scaled to a mean of zero and a standard deviation of 1. We then fitted null models using ‘time' as a smooth term (a thin-plate regression spline) and ‘pond' as a random effect (a factor-smooth interaction with time). Effects of treatments were explored by adding fixed effects to the null model and comparing models using AIC. Statistical significance of treatments in the model with the lowest AIC was verified using the summary.gam() function. We also plotted fitted values and their 95% confidence intervals to assess the time-dependence of treatment effects. Model selection results are provided in electronic supplementary material, table S1, and fitted values with confidence intervals are shown in electronic supplementary material, figures S1 and S2.

We also fitted GAMMs using raw (untransformed) values of chlorophyll a and crustacean density to compare experimental results with the output of our theoretical model. These GAMMs used a Tweedie distribution for the residuals to account for the zero-inflated, positive and right-skewed distribution of density values. Chlorophyll *a* and crustacean density were rescaled between 0 and 1 so that they could be plotted on a common axis. All pH and dispersal treatments were pooled. Models included ‘time' as a fixed effect and ‘pond' as a random effect. The output of these GAMMs is shown in figure 5.

## 4. Results

### (a) Metacommunity structure

Our experiment did not detect any consistent effect of dispersal, which we had confidently expected on the basis of microcosm experiments (see electronic supplementary material, figure S1 and table S1). We speculate that dispersal is a double-edged treatment in trophically structured communities: it supplies potentially adapted immigrants to a stressed community, but it also supplies predators, including types resistant to stress. If these opposed effects are similar in magnitude, it might be difficult to detect any overall effect of dispersal with the levels of replication we were able to achieve. Because we found no effect of dispersal, we have pooled dispersal treatments to evaluate the effect of prior stress.

### (b) Phytoplankton

**Figure 4.**
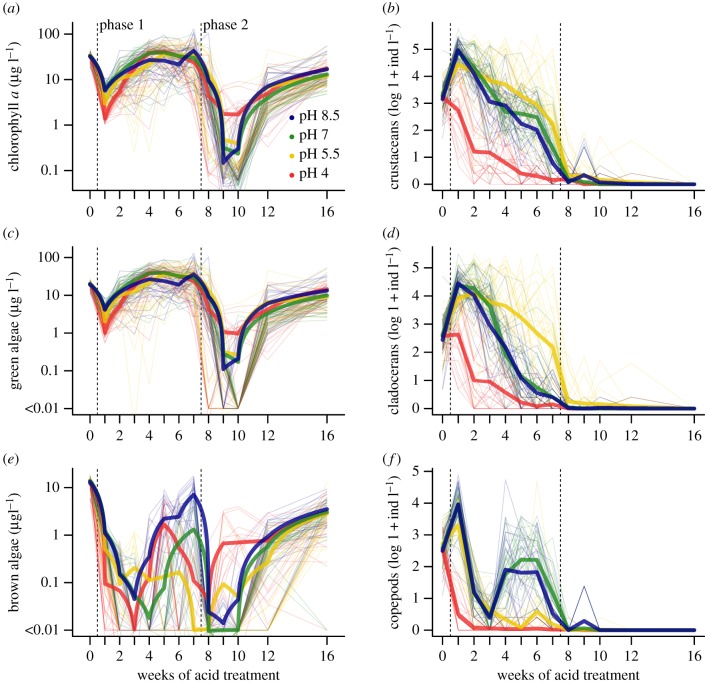
Dynamics of the plankton over the course of the experiment. (*a*,*c*,*e*) Time series of phytoplankton biomass for (*a*) total biomass, (*c*) green algae, (*e*) brown algae (diatoms, chrysophytes and dinoflagellates). (*b*,*d*,*f*). Time series of crustacean zooplankton density for (*b*) total density, (*d*) cladocerans, (*f*) copepods. Each line represents a single mesocosm over time. The thick coloured lines are averages for the four pH treatments. (Online version in colour.)

The dynamics of the phytoplankton are shown in figure 4*a* (overall), *c* (chlorophytes) and *e* (brown algae, mostly diatoms and dinoflagellates). The founding phytoplankton community was dominated by chlorophytes (59.4%) and browns (39.9%), with minor contributions from cryptophytes (0.6%) and cyanobacteria <0.1%). During phase 1 chlorophytes increased in frequency (90.0%) while browns declined (9.4%), and cryptophytes (0.1%) and cyanobacteria (0.5%) remained rare. Browns were most abundant at pH 8.5 late in phase 1. During phase 2, the community was again dominated by chlorophytes (85.7%), although browns (13.3%) became somewhat more frequent; cryptophytes (less than 0.1%) and cyanobacteria (1.0%) remained rare.

In the week following the inoculation of the mesocosms phytoplankton density fell sharply to about 10% of its initial value (figure 4*a*). There was a strong effect of pH, with the communities of more acidified mesocosms being less abundant (GAMM, interaction of time and pH: *p* < 0.001; electronic supplementary material, table S1 and figure S2). Over the next three weeks, the communities recovered and the effect of pH weakened and became less consistent. By the end of phase 1, average abundance was restored to its pre-treatment level and there was no consistent effect of acidification (figure 4*a*; electronic supplementary material, figure S2).

At the onset of phase 2, all communities at first collapsed to very low abundance, with about 98% of phytoplankton being removed by severe acidification, and then began to recover over the next two weeks. The collapse was less marked for those communities that had been strongly acidified to pH 4 in phase 1 (electronic supplementary material, figure S2). By the end of phase 2, all communities had recovered and average abundance had increased by a factor of about 20, although it was only about half the value at the end of phase 1. Final chlorophyll a concentration in recovered ponds was comparable with its value in untreated (control) ponds (electronic supplementary material, figure S3).

### (c) Zooplankton

The dynamics of the crustacean zooplankton are shown in figure 4*b* (overall), *d* (cladocerans) and *f* (copepods). The crustacean zooplankton initially comprised cladocerans and copepods in almost equal abundance (51.5% and 48.5%, respectively). During phase 1, cladocerans became more frequent (78.5%) and copepods declined (21.5%). Cladocerans were more frequent at pH 5.5 than at any other level; copepods became abundant at higher pH late in phase 1.

In the week following the inoculation of the mesocosms, the abundance of crustacean zooplankton (cladocerans and copepods) increased to about four times its original value. There was a strong effect of pH, with peak abundance in the order of pH treatment, the most highly acidified mesocosms (pH 4) declining slightly (GAMM, interaction effect of time and pH: *p* < 0.001; electronic supplementary material, table S1 and figure S2). In the following weeks, zooplankton abundance declined to about its original value by the end of phase 1, except in the most highly acidified mesocosms, where zooplankton were almost entirely eliminated.

At the onset of phase 2, the zooplankton collapsed to very low abundance in all mesocosms and never recovered. Occasional animals were found during phase 2, but no viable community became established in any of the mesocosms. By contrast, crustaceans persisted in control ponds that were not acidified in phase 2 (electronic supplementary material, figure S3), showing that the collapse in acidified ponds cannot be attributed to a seasonal effect.

## 5. Discussion

The shifts in abundance of phytoplankton and zooplankton during phase 1 of our experiment, and the rescue of phytoplankton in phase 2, can be understood in general terms as the outcome of severe physical stress in a trophically structured community. The plankton community dynamics of our mesocosms may, in some degree, reflect a natural succession, for example the increase of copepods at lake pH in midsummer, although in other respects, for example the absence of any cyanobacterial bloom, it differed from the lake from which the experimental communities were derived (see [24]).

### (a) Phase 1

Crustacean zooplankton in the mesocosms are released from fish predation and immediately increase in abundance unless prevented from doing so by acidification. The phytoplankton community collapses through the combination of increased predation and physical stress. These work in opposite directions because the zooplankton are more sensitive to acidification than the phytoplankton (a stressor with different trophic selectivity, such as a herbicide, might have quite different consequences). Shortage of prey then causes the zooplankton to decline and the phytoplankton recover; recovery is fastest in the most highly acidified mesocosms, from which zooplankton have been almost completely removed. By the middle of phase 1, the initial effect of pH has disappeared because the effects of physical stress and predation are roughly balanced. By the end of phase 1, the community as a whole is similar in overall abundance and gross composition to its initial state, except in the most highly acidified mesocosms.

### (b) Phase 2

The collapse of the phytoplankton community under lethal stress shows a clear signal of prior exposure to a sublethal level of the same stressor: communities that have previously been exposed to pH 4 decline less. The collapse of the zooplankton community, however, is irreversible, and the elimination of predators permits the later rescue of phytoplankton communities with a history of less severe (or no) stress. This is consistent with the conclusion of Thibodeau *et al*. [14] that ‘Reduced zooplankton grazing pressure represents an additional effect of pH perturbation to aquatic ecosystems that could enable the ecological response observed in phytoplankton communities.' There is some change in the composition of the phytoplankton community from phase 1 to phase 2, but the rank order of major groups remains the same. An alternative explanation is that tolerance of low pH and resistance to predation are positively correlated. At low pH during phase 1, the zooplankton are scarce, and the recovery of phytoplankton in phase 2 is attributable to their prior exposure to pH stress; at high pH during phase 1 zooplankton are abundant and select for inedible phytoplankton, which subsequently recover in phase 2 because they are also resistant to high pH. We intend to obtain more detailed data on the composition of the phytoplankton community to evaluate this more complex hypothesis.

**Figure 5.**
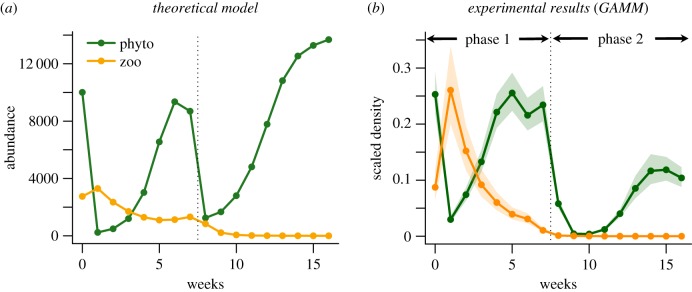
Comparison of the output of (*a*) a modified Lotka–Volterra model with (*b*) the observed dynamics (GAMM fit with 95% confidence interval when pooling all pH treatments). Abundance in the model is nominally in units of individuals, but could refer to density in arbitrary units; abundance is rounded to an integer, so that very small populations (less than 1 nominal individual) become extinct, which seems reasonable when using these relations to represent rescue. There is no explicit cost of resistance in these equations, but I have assumed that resistant types will remain rare unless the community experiences severe stress. Parameter values were: *a* 0.5, *d* 0.3, × 0.0005, *c* 0.1, *s_P_* 0.8, *r_P_* 0, *s_Z_* 0.5, *r_Z_* 0, *Q* 10 000. The frequency of resistant types in the phytoplankton under mild stress in the model was about 10% at the end of phase 1 and had reached about 95% late in phase 2. (Online version in colour.)

These conclusions can be expressed in a more quantitative fashion by comparing our observations with the simple predator–prey model described above (figure 5). The model is intended to illustrate our hypothesis rather than to provide a detailed numerical account of dynamics; parameter values were not independently estimated, but rather chosen such that both phytoplankton and zooplankton persisted through phase 1. With this reservation in mind, the model output shows that the dynamics we observed can be understood in terms of the effect of physical stress modulated by predator–prey interaction. In phase 1, the coupled dynamics of phytoplankton and zooplankton, and the rapid recovery of phytoplankton in stressful treatment where zooplankton have declined, are both captured by the model. In phase 2, the collapse of the zooplankton and the subsequent recovery of phytoplankton are also captured by the model. The rescue of the phytoplankton community in the model is attributable, as expected, to the spread of types resistant to physical stress.

Our knowledge of community composition is limited to the major categories of green/brown algae and cladoceran/copepod crustacean zooplankton. A more complete explanation of community dynamics would require more detailed information, but with limited resources there is inevitably a trade-off between a well-replicated study with low taxonomic resolution and a poorly replicated study with high taxonomic resolution. To investigate broad principles, we chose the former approach and conducted 1248 surveys of the mesocosms (96 mesocosms × 13 dates), necessarily at low taxonomic resolution. Even so, we were surprised by the high degree of variation among replicate mesocosms inoculated from the same source and subjected to the same combination of treatments. A fully satisfactory experiment, combining high replication with high taxonomic resolution, might require a level of funding comparable with large projects in particle physics, astronomy or genomics.

Our experiment confirms the leading contribution of prior exposure to rescue under lethal physical stress and to this extent justifies the extrapolation of the results of microcosm experiments to mesocosm scale. At mesocosm scale, however, the community dynamics we observed and the output of the model suggest that the rescue of the phytoplankton appears to be conditional on release from predation because of the more severe effect of the stressor on the zooplankton. The effect of the physical stress is thus modulated by biotic interactions in a trophically structured community.

## Supplementary Material

Details of Generalizable Additive Mixed Model analysis.

## Data accessibility

The primary data for this experiment can be found in the electronic supplementary material and are available from the Dryad Digital Repository: https://doi.org/10.5061/dryad.7qt30vf [25].

## References

[1] Bell G, Gonzalez A 2009 Evolutionary rescue can prevent extinction following environmental change. Ecol. Lett. 12, 942–948. (10.1111/j.1461-0248.2009.01350.x) 19659574

[2] Bell G, Gonzalez A 2011 Adaptation and evolutionary rescue in metapopulations experiencing environmental deterioration. Science 332, 1327–1330. (10.1126/science.1203105) 21659606

[3] Gonzalez A, Bell G 2012 Evolutionary rescue and adaptation to abrupt environmental change depends upon the history of stress. Phil. Trans. R. Soc. B 368, 20120079 (10.1098/rstb.2012.0079) PMC3538446 23209161

[4] Lindsey HA, Gallie J, Taylor S, Kerr B 2013 Evolutionary rescue from extinction is contingent on a lower rate of environmental change. Nature 494, 463–467. (10.1038/nature11879) 23395960

[5] Govaert L, Pantel JH, de Meester L 2016 Eco-evolutionary partitioning metrics: assessing the importance of ecological and evolutionary contributions to population and community change. Ecol. Lett. 19, 839–853. (10.1111/ele.12632) 27339378

[6] van Benthem KJ, Bruijning M, Bonnet T, Jongejans E, Postma E, Ozgul A 2017 Disentangling evolutionary, plastic and demographic processes underlying trait dynamics: a review of four frameworks. Methods Ecol. Evol. 8, 75–85. (10.1111/2041-210X.12627)

[7] Low-Décarie E, Kolber M, Homme P, Lofano A, Dumbrell A, Gonzalez A, Bell G 2015 Community rescue in experimental metacommunities. Proc. Natl Acad. Sci. USA 112, 14 307–14 312. (10.1073/pnas.1513125112) 26578777 PMC4655536

[8] Kovach-Orr C, Fussmann GF 2013 Evolutionary and plastic rescue in multitrophic model communities. Phil. Trans. R. Soc. B 368, 20120084 (10.1098/rstb.2012.0084) 23209166 PMC3538451

[9] Yamamichi M, Miner BE 2015 Indirect evolutionary rescue: prey adapts, predator avoids extinction. Evol. Appl. 8, 787–796. (10.1111/eva.12295) 26366196 PMC4561568

[10] Fussmann GF, Gonzalez A 2013 Evolutionary rescue can maintain an oscillating community undergoing environmental change. Interface Focus 3, 20130036 (10.1098/rsfs.2013.0036) 24516721 PMC3915851

[11] Northfield TD, Ives AR 2013 Coevolution and the effects of climate change on interacting species. PLoS Biol. 11, e1001685 (10.1371/journal.pbio.1001685) 24167443 PMC3805473

[12] Osmond MM, Otto SP, Klausmeier CA 2017 When predators help prey adapt and persist in a changing environment. Am. Nat. 190, 83–98. (10.1086/691778) 28617633

[13] Tseng M, O'Connor MI 2015 Predators modify the evolutionary response of prey to temperature change. Biol. Lett. 11, 20150798 (10.1098/rsbl.2015.0798) 26673935 PMC4707700

[14] Thibodeau G, Walsh DA, Beisner BE 2015 Rapid eco-evolutionary responses in perturbed phytoplankton communities. Proc. R. Soc. B 282, 20151215 (10.1098/rspb.2015.1215) PMC4571698 26311667

[15] Findlay DL, Kasian SEM 1990 Phytoplankton communities of lakes experimentally acidified with sulfuric and nitric acids. Can. J. Fish. Aquat. Sci. 47, 1378–1386. (10.1139/f90-157)

[16] Yan ND 1979 Phytoplankton community of an acidified, heavy metal-contaminated lake near Subury, Ontario: 1973–1977. Water Air Soil Pollut. 11, 43–55. (10.1007/BF00163517)

[17] Hendrey GR, Baalsrud K, Laake M, Raddum G 1976 Acid precipitation: some hydrobiological changes. Ambio 5, 224–227.

[18] Havens K, DeCosta J 1985 An analysis of selective herbivory in an acid lake and its importance in controlling phytoplankton community structure. J. Plankton Res. 7, 207–222. (10.1093/plankt/7.2.207)

[19] Nicholls KH, Nakamoto L, Keller W 1992 Phytoplankton of Sudbury area lakes (Ontario) and relationships with acidification status. Can. J. Fish. Aquat. Sci. 49(Suppl 1), 40–51. (10.1139/f92-299)

[20] Keller W, Gunn JM, Yan ND 1992 Evidence of biological recovery in acid-stressed lakes near Sudbury, Canada. Environ. Pollut 78, 79–85. (10.1016/0269-7491(92)90013-Z) 15091931

[21] Gray DK, Arnott SE 2009 Recovery of acid-damaged zooplankton communities: measurement, extent and limiting factors. Environ. Rev. 17, 81–99. (10.1139/A09-006)

[22] R Core Team. 2018 R: a language and environment for statistical computing. Vienna, Austria: R Foundation for Statistical Computing.

[23] Wood SN 2017 Generalized additive models: an introduction with R. London, UK: Chapman & Hall, CRC.

[24] Kalff J 1972 Net plankton and nanoplankton production and biomass in a North Temperate zone lake. Limnol. Oceanogr. 17, 712–720. (10.4319/lo.1972.17.5.0712)

[25] Bell G, Fugère V, Barrett R, Beisner B, Cristescu M, Fussmann G, Shapiro J, Gonzalez A 2019 Data from: Trophic structure modulates community rescue following acidification Dryad Digital Repository. (10.5061/dryad.7qt30vf) PMC6571482 31185868

