# Supplementary material for: Trophic structure modulates community rescue following acidification
Source: Proc Biol Sci. 2019 Jun 12;286(1904):20190856. doi: 10.1098/rspb.2019.0856 (PMC6571482; doi:10.1098/rspb.2019.0856)

Table S1. Results of GAMM model selection showing, for each model, the mgcv model syntax, the model AIC, and the difference in AIC (Δ AIC) with the best-fitting model, including only a pH effect. 'Structure' refers to metacommunity structure, i.e. homogeneous vs. heterogeneous metacommunities. All models included a random factor-smooth interaction between 'pond' and 'time': s(time, pond, bs='fs'). Disp. = dispersal. Str. = structure.

**Model name**	**Fixed effects** (mgcv syntax)	**AIC**	**Δ AIC**
Phytoplankton: response variable = chlorophyll a *concentration (log-transformed)*			
Null model	s(time)	3450	246
pH only model	pH + s(time, by = pH)	3204	0
Dispersal only model	disp + s(time, by = disp)	3470	266
Structure only model	str + s(time, by = str)	3457	253
pH by dispersal model	pH + disp + s(time, by = pH) + s(time, by = disp) + s(time, by = interaction(pH,disp))	3228	24
pH by structure model	pH + str + s(time, by = pH) + s(time, by = str) + s(time, by = interaction(pH,str))	3236	32
Three-way interaction	pH + disp + str + s(time, by = pH) + s(time, by = disp) + s(time, by = str) + s(time, by = interaction(pH,disp,str))	3230	26
Zooplankton: response variable = crustacean density (log(1+x)-transformed)			
Null model	s(time)	2971	302
pH only model	pH + s(time, by = pH)	2669	0
Dispersal only model	disp + s(time, by = disp)	2979	310
Structure only model	str + s(time, by = str)	2982	313
pH by dispersal model	pH + disp + s(time, by = pH) + s(time, by = disp) + s(time, by = interaction(pH,disp))	2677	8
pH by structure model	pH + str + s(time, by = pH) + s(time, by = str) + s(time, by = interaction(pH,str))	2673	4
Three-way interaction	pH + disp + str + s(time, by = pH) + s(time, by = disp) + s(time, by = str) + s(time, by = interaction(pH,disp,str))	2690	21

Figure S1. Fitted values with 95 % confidence bands for GAMMs including either phytoplankton or zooplankton abundance as the response, time as a smooth term, and pH and dispersal mode as factors and factor-smooth interactions. These fitted values correspond to the 'pH by dispersal' models described in Table S1. Dispersal mode had no significant effect on plankton abundance over time nor its response to pH treatments.

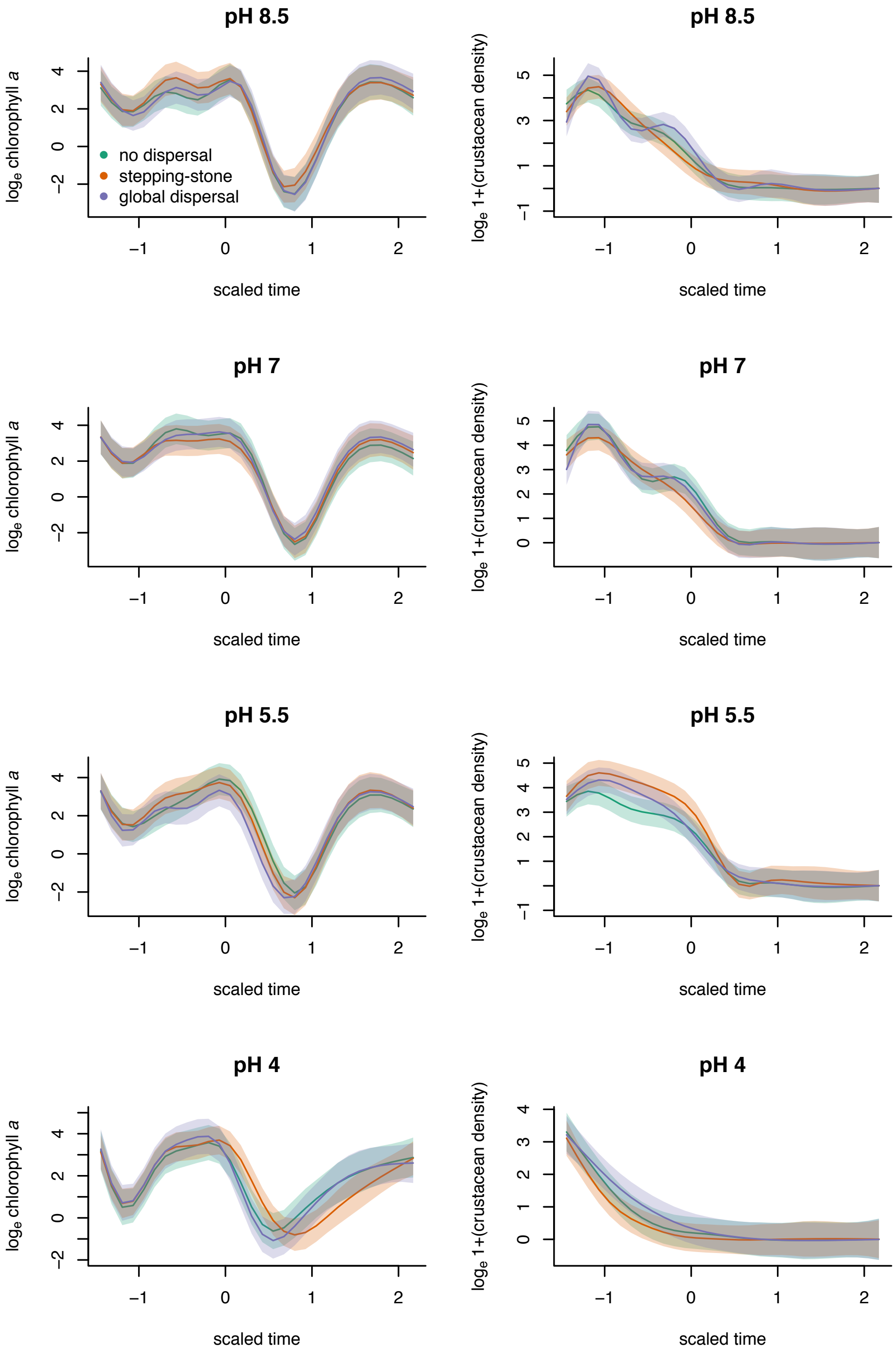

Figure S2. Fitted values with 95 % confidence bands for GAMMs including either phytoplankton biomass (a) or zooplankton density (b) as the response, time as a smooth term, and pH as a factor and a factor-smooth interaction. These fitted values correspond to the 'pH only' models described in Table S1. pH had a significant, time-dependent effect on plankton abundance.

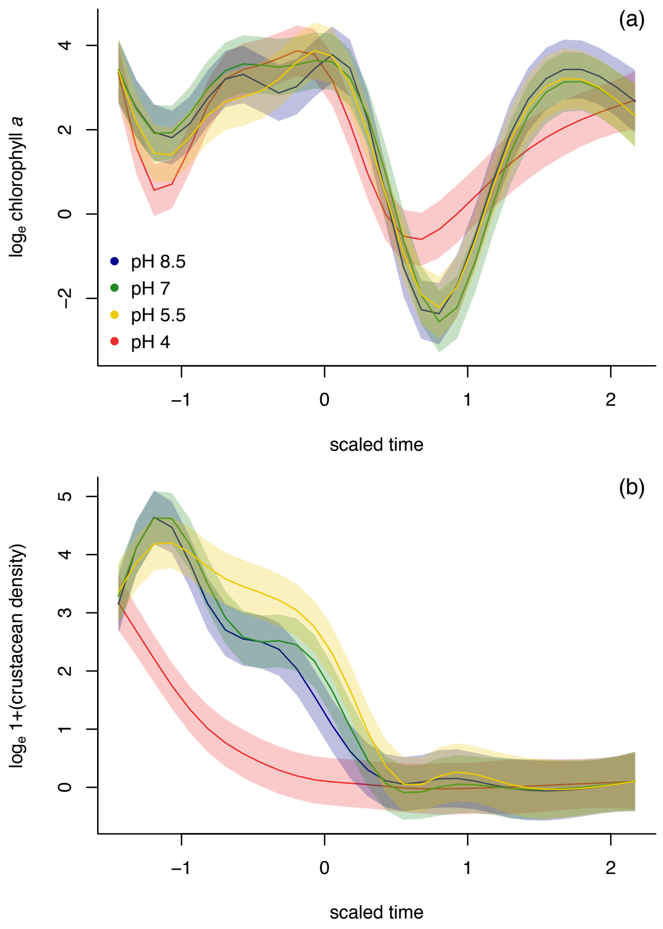

Figure S3. Phytoplankton biomass (a) and zooplankton density (b) in all ponds at the end of Phase 2, including the 4 control ponds ('C') which were not acidified in Phase 2. Acidified ponds are grouped based on pH treatment in Phase 1.

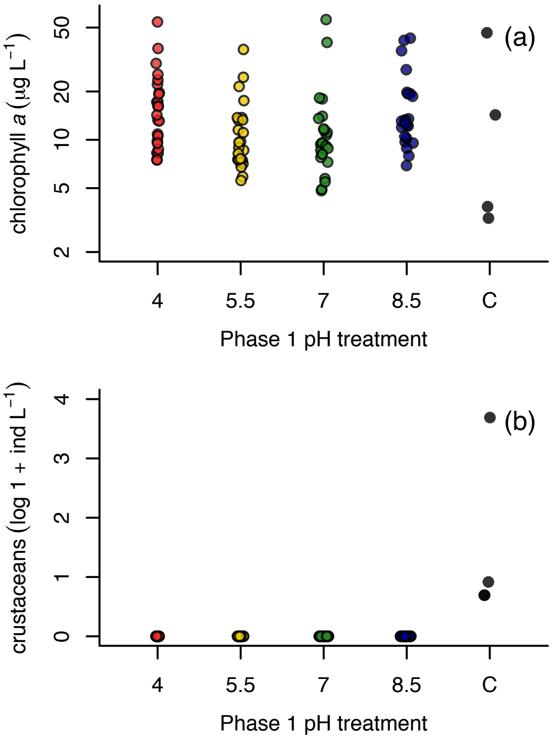

Supplement: Details of Generalizable Additive Mixed Model analysis. [file rspb20190856supp1.pdf]
